# Assessment of Current Malaria Status in Light of the Ongoing Control Interventions, Socio-Demographic and Environmental Variables in Jiga Area, Northwest Ethiopia

**DOI:** 10.1371/journal.pone.0146214

**Published:** 2016-01-11

**Authors:** Seble Ayalew, Hassen Mamo, Abebe Animut, Berhanu Erko

**Affiliations:** 1 Department of Microbial, Cellular and Molecular Biology, College of Natural Sciences, Addis Ababa University, P.O. Box 1176, Addis Ababa, Ethiopia; 2 Aklilu Lemma Institute of Pathobiology, Addis Ababa University, P.O. Box 1176, Addis Ababa, Ethiopia; Quensland University of Technology, AUSTRALIA

## Abstract

Following substantial decline in malaria burden in Ethiopia, the country is planning to eliminate malaria in certain low transmission settings by 2020. To evaluate the attainability of this goal in-depth examination of malaria parasite carriage at community level is necessary. This study was, therefore, aimed at assessing the current situation of malaria in relation to ongoing control interventions in Jiga area, Jabi Tehnan District in northwest Ethiopia. A cross-sectional household (HH) survey was conducted in November-December 2013. Out of 2,574 HHs (11,815 people) in the entire Jabi Tehnan District, 392 (accommodating 1911 people) were randomly selected from three purposely selected villages. One randomly selected member from each selected HH was tested for malaria using rapid diagnostic test (mRDT). All participants tested for malaria (n = 392) were afebrile (axillary temperature <37.5°C). Eleven individuals (2.8%, 95% confidence interval (CI):1.2–4.4%) were found to be mRDT positive. Most HHs (95.9%, 95% CI: 93.5–97.5%) had at least 1 long-lasting insecticidal net (LLIN). Insecticide residual spraying (IRS) coverage the last six months was 85.5% (95% CI: 82.0–88.9%). Malaria prevalence remains unexpectedly high despite high HH coverage of control interventions.

## Introduction

Prompt case detection and treatment with artemisinin-based combination therapies, use of insecticide treated nets (ITNs) and indoor residual spraying (IRS) resulted in reduction of malaria related cases and deaths globally [1, 2]. In Ethiopia, prior to 2004 malaria was known to cause 5–10 million clinical cases and 70,000 deaths each year [3]. Currently, however, the figures are much lower [4] because of the country’s scale-up of its control interventions in line with the global malaria control initiative. Between 2004 and 2012, nearly 46 million long-lasting insecticidal nets (LLINs) were distributed, over 70% of targeted households (HHs) covered with IRS and 9 million doses of malaria treatment provided to public health facilities all free of charge [5]. Encouraged by its achievement the Federal Ministry of Health (FMoH) aims to achieve malaria elimination within specific geographical areas with historically low malaria transmission and zero deaths due to malaria in areas with malaria transmission by 2020 [4].

Nevertheless, in the Amhara Region, where the initial malaria elimination project is planned in selected Districts, 73.7% of the HHs had at least one net in 2011 which was slightly lower compared to the level in 2007 (75.2%) and malaria slide positivity has increased from 0.6% (in 2007) to 2.0% in 2011 [6]. A total of 1,127,241 cases of malaria were reported in this Region in 2012, out of a population of 19,867,817 habitants [5]. West Amhara which consists of five administrative zones (the Amhara Region is subdivided into 11 administrative zones including Bahir Dar special zone) has accounted for 93.1% of the Region’s malaria burden of which West Gojjam had the greatest number of cases (404,926) followed by North Gondar (225,818). Jiga area which is located in Jabi Tehnan District, West Gojjam Zone, northwest Ethiopia, is among the top malaria affected areas in the Region deserving particular attention. Out of 194,818 patients examined for malaria between September 2009 and August 2013 at Jiga Health Center, 24,103 (25.4%) had slide-confirmed malaria [7]. A study conducted five years back also revealed high malaria prevalence (65.4%) among febrile patients in Jiga [8].

Thus, it is necessary to assess the current prevalence of malaria and coverage of control interventions in Jiga area. This study, therefore, assessed malaria prevalence and coverage of control interventions in this area. Ownership/utilization of LLINs and coverage of IRS; and socio-economic, environmental and demographic factors were captured and analyzed in relation to malaria status. The baseline information produced can be utilized by concerned health professionals and policy makers for malaria control and possible elimination in the area.

## Materials and Methods

### Study area description

**Fig 1:**
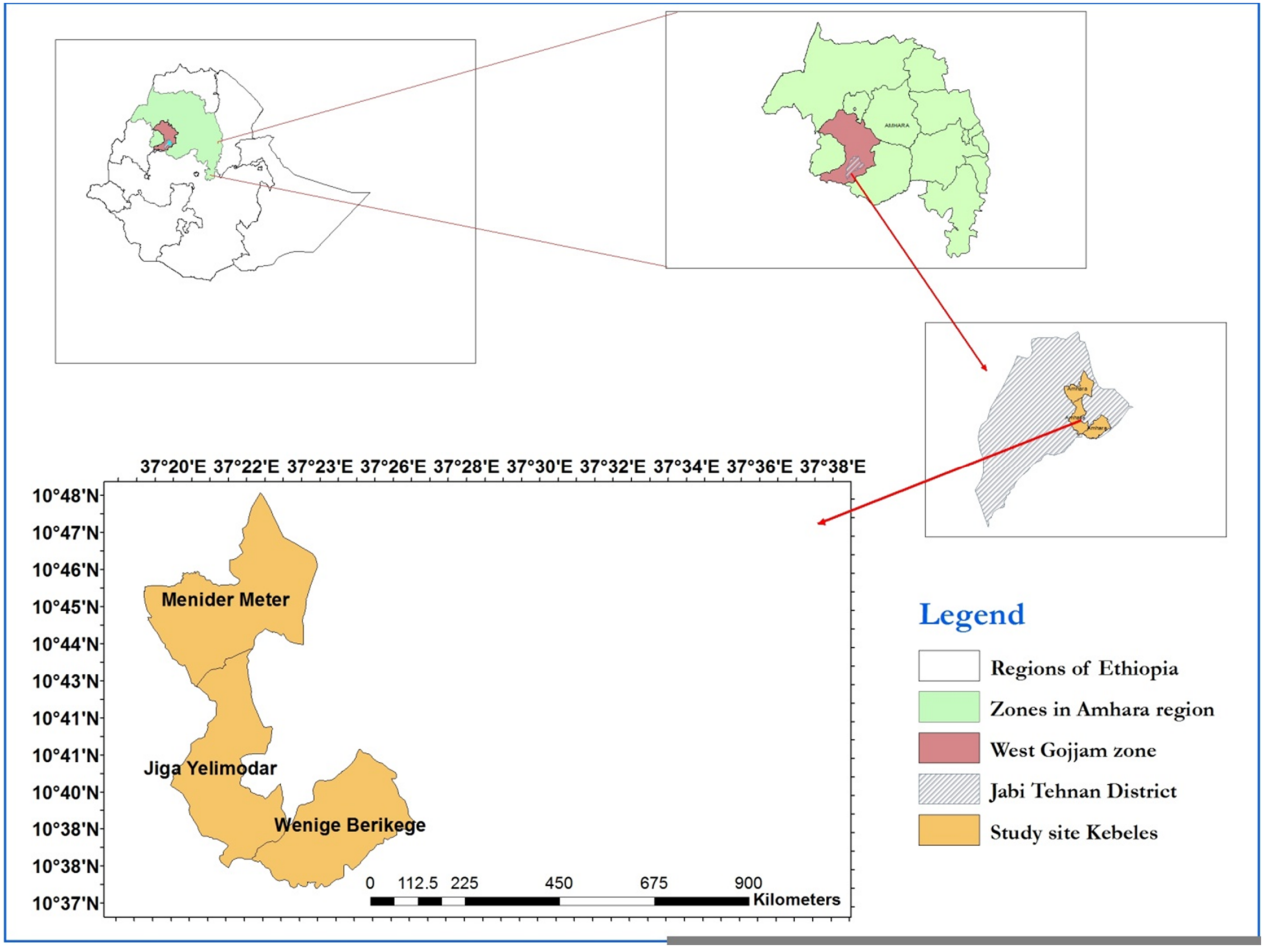
Map of Ethiopia and the surveyed households in Jiga area, northwest Ethiopia.

The study was conducted in Jiga area, northwest Ethiopia, which is located at 372 km from Addis Ababa (Fig 1). The area is situated at 9°20' and 14°20' north latitude and 36°20' and 40°20' east longitude, and 1812 meters above sea level with an area of 1169.54 km^2^ [9,10]. It was selected purposely as it is one of malaria hotspots in the Zone. The mean annual temperature is about 23°C (ranging from 14°C to a slightly above 32°C) and the mean annual rainfall is 1250 millimeter. Agriculture is the principal source of livelihood for the population. Cereal crops such as *teff* (*Eragrostis tef*), maize, wheat and barley are the most commonly cultivated crops in the District. Pepper, sugarcane and coffee are cash crops in some sites of the District including the study villages (*kebeles*). In the study area, malaria transmission peaks bi-annually from September to December, and April to May following the major rainy season and small rains, respectively.

### Study design, population and sampling

The study was a cross-sectional HH survey and conducted in November-December 2013. The minimum number of the study participants was estimated using minimum sample size determination formula n = z^2^p(1-p)/d^2^ [11]; where n = the sample size, z = 1.96 at 95% confidence interval (IC), d = margin of error, p = expected malaria prevalence rate in the locality which was assumed to be 50% with 95% CI and margin of error (d) at 5% (standard value of 0.05). Then n was computed to be 384. With a non-response rate of 2% the total sample size was 392.

To arrive at sampling HHs, first a list of forty-one clusters (*kebeles =* villages) was obtained from the District administration from which three malarious villages were selected based on ease of accessibility. The estimated sample size was proportionally distributed to the selected three villages based on their size and HHs were randomly chosen.

### Data collection

The lottery method was used to randomly select any (one year and older) single HH member for rapid malaria screening using SD FK80 Pf/Pv malaria antigen rapid test (Standard diagnostics, Korea) as per the manufacturer’s instruction. To ensure maximum participation, HHs with absentees were revisited a second time on the same day to get those missing at the first visit. A structured and pre-tested questionnaire was administered to gather HH, individual and environmental variables.

Questionnaire data was reviewed and checked for completeness, accuracy and consistency during and at the end of each day of data collection. Observationally verifiable respondents’ responses pertaining to IRS, presence, number and type and status (as per the World Health Organization criteria) of mosquito nets; demographic information; and other related items were verified by the first author. Data were coded (de-identified) and entered into separate Microsoft Office Excel spreadsheet, cleaned, edited and exported to statistical package for social sciences version 20.0 for Windows (IBM SPSS Statistics, USA) for analysis. Intra-HH sampling probabilities were calculated and important individual variables (age, sex, mRDT positivity and LLIN use previous night) were weighted for HH size.

Univariate logistic regression analysis was used to examine the association between each potential risk factor and mRDT positivity. A multivariate model was then constructed by backward stepwise logistic regression analysis. The cutoff for retention of a variable in the model was set at p≤0.1. All potentially significant variables were entered in the model and explanatory variables were sequentially removed when the exit cutoff is satisfied. Age and LLIN use the previous night were kept in all multivariate models, irrespective of statistical significance, to control for any confounding effects. *P≤*0.05 was considered statistically significant.

### Ethical considerations

The study was conducted after obtaining ethical clearance from College of Natural Sciences Institutional Ethics Review Board, Addis Ababa University. A written informed consent was sought for adults and parents consented for children. Qualified local health post workers collected blood samples and treated malaria positive cases, free of charge, as per the national guideline.

## Results

### Socio-demographic characteristics

**Fig 2:**
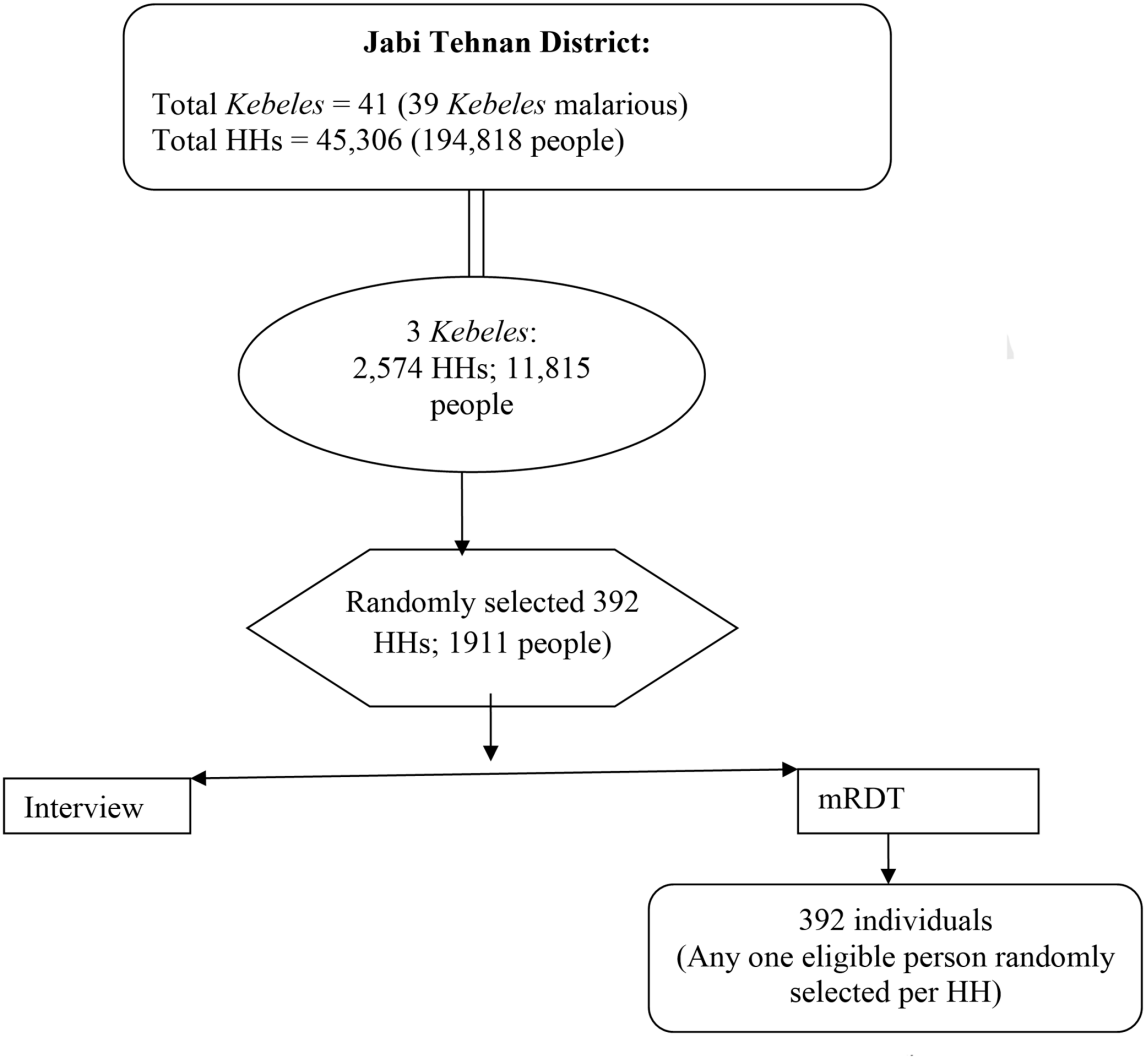
Flow diagram of household and individual participants enrolled for malaria screening.

All visited HHs and selected individual members were willing to participate in the study. A total of 392 HHs (housing 1911 people) were visited in the three villages. One hundred and sixty-nine (43.1%) of the HHs had a family size of 1–4, 188(48.0%) 5–7 and 35(8.9%) had ≥8 persons per HH. The mean HH size was 4.9(95% CI: 4.7–5.1). Totally 392 individuals, one person from each HH, were tested for malaria (Fig 2). Among these, 48 (12.2%) individuals were 1–4.9, 117(29.8%) 5–14 and 227(57.9%) were ≥15 years old. The maximum age was 80 years. The mean age was 21.9 year (95% CI: 20.3–23.6). The number of males was 149 and there were 243 female participants. Out of 158 women of childbearing age 14(8.8%) were self-reportedly pregnant. All individual factors described herewith are crude and refer to mRDT-tested subjects unless otherwise specified. Weighted and crude results are displayed side by side, for individual factors, in tables 1 and 2.

**Table 1:** Malaria prevalence in the three villages surveyed in Jiga using mRDT (*P*. *falciparum* and *P*. *vivax* combined and weighted for HH size).

Village	N		no, % (95% CI)	
	crude	weighted	crude	weighted
*Wenige Berikege*	92	3257	3, 3.3(1.1–9.2%)	121, 3.7(3.1–4.4%)
*Jiga Yelimodar*	131	4521	1, 0.8% (0.1–4.2%)	50, 1.1(0.8–1.5)
*Menider Meter*	169	5871	7, 4.1(2.0–8.3)	279, 4.7(4.2–5.3)
Total	392	13649	11, 2.8(1.6–4.9)	450, 3.3(3.0–3.6)

**Table 2:** Univariate and multivariate logistic regression analysis of individual, HH and environmental risk factors for mRDT positivity (individual factors weighted for HH size).

Variable	n		mRDT pos (no, %)		Univariate analysis		Multivariate analysis	
	crude	weighted	crude	weighted	OR (95% CI)	P-value	OR (95% CI)	P-value
Age (year)								
1–4.9	48	1879	2(4.1)	79(4.2)	2.13(0.35–13.14)	0.415	2.56(0.64–10.21)	0.183
5–14.9	119	4450	4(3.4)	200(4.5)	1.70(0.37–7.77)	0.491	2.24(0.38–13.15)	0.371
≥15	225	7321	5(2.2)	171(2.3)	1.00		1.00	
Sex								
Male	149	5407	3(2.0)	129(2.4)	0.60(0.16–2.31)	0.461	-	-
Female	243	8243	8(3.3)	321(3.9)	1.00			
Has slept under LLIN last night	392	13221						
Yes	286	10264	10(3.5)	400(3.9)	1.00		1.00	
No	106	3386	1(0.94)	50(1.5)	0.31(0.24–2.46)	0.267	0.30(0.04–2.48)	0.262
LLIN/HH ratio	392							
≥0.5	98	-	6(6.1)	-	0.28(0.08–0.94)	0.04	0.27(0.08–0.92)	0.036
<0.5	294	-	5(1.7)	-	1.00		1.00	
HH proximity to mosquito breeding site	392	-						
≥1000m	380	-	9(2.4)	-	0.12(0.02–0.64)	0.013	0.12(0.02–0.64)	0.014
<1000m	12	-	2(16.7)	-	1.00		1.00	
Insecticide sprayed ≤3 months ago	392	-						
Yes	105	-	1(.95)	-	3.76(0.46–29.7)	0.210	-	-
No	287	-	10(3.5)	-	1.00			

### LLIN and IRS coverage

Three hundred seventy six (95.9%, 95% CI: 93.5%-97.5%) HHs owned at least one LLIN. Hundred thirty eight (36.7%) HHs owned more than 2 LLINs, 204(54.3%) two and 34(9.0%) owned only one. The mean number of LLINs owned per HH was 1.8 (95% CI: 1.7414–1.8913). The LLIN to HH ratio was below 0.5 for 278 HHs, that is only 98 HHs (26.1%, 95% CI: 21.7–30.5%) had at least one LLIN for every two people. When HHs with 0 LLIN were merged with these 278, the total number of HHs with LLIN/HH ratio below 0.5 was 294 (75%). However, the proportions of children under 5 years old and pregnant women with access to LLINs within their HHs (having earmarked LLINs) were 97.9% (95% CI: 89.1–99.6) and 95.2% (95% CI: 77.3–99.2), respectively. The LLINs were distributed to 348(92.5%) of the HHs in the previous two months prior to this survey, while 28(7.4%) HHs acquired the LLINs some three years back.

The reason mentioned for lack of LLINs during the study period, by all the HHs that lacked the LLINs, was the problem of getting substitutes for worn out ones. Regarding the condition of the LLIN possessed by the participants, 373(99.0%) were graded as in good conditions and 3(0.8%) were not. Among pregnant women 71.4% (95% CI: 64.4–78.5%) used LLINs the previous night. Overall, among individuals who belonged to LLIN-owning HHs 286(76.1%, 95% CI: 71.9–80.4%) slept under LLIN the previous night. While the proportion of children under-5 years old who slept under LLIN the previous night was 77.1% overall, it was 78.7% for those who belonged to LLIN-owned HHs. The reasons for not using the LLINs were that 33(36.7%) HHs had no beds and 57(63.3%) due to incompatible house structures that could not allow LLIN use.

The overall IRS coverage in the study locality was 85.5% (95% CI: 82.0–88.9%). Of these, 105 HHs (31.3%, 95% CI: 22.4–40.2%) were sprayed in the last 3 months of the study year and 230(68.6%, 95% CI: 62.6–74.6%) were sprayed in the last six months.

### Malaria parasite prevalence

Overall, 11(2.8%, 95% CI: 1.2–4.4%) individuals were mRDT positive, males and females being 3(2%) and 8(3%) respectively. Three cases were from *Wongie berikegn* village (n = 92), 7 from *Menider meter* (n = 169) and 1 from *Jiga yelimodar* (n = 131) (Table 1). *P*. *falciparum* and *P*. *vivax* were detected in 6 and 5 individuals, respectively. Among *Wongie berikegn* participants, 1 person had *P*. *falciparum* infection and 2 were positive for *P*. *vivax* infection. In *Mendier meter*, 5 of the cases were *P*. *falciparum* and 2 were *P*. *vivax*. The single infected case from *Jiga yelimodar* was *P*. *vivax*. There appeared to be no variation in the prevalence of malaria o between sex and age groups as well as study villages. But this was not tested statistically due to the small number of mRDT positives. None of the 14 pregnant women was mRDT positive.

In univariate analysis HHs located at ≥1000m from mosquito breeding sites were at significantly lower risk of mRDT positivity in comparison with those that were at a shorter distance (odds ratio (OR) = 0.12, 95% CI: 0.02–0.64, p = 0.013) (Table 2). HHs having LLIN/HH size ratio ≥0.5 had significantly lower (0.0%) mRDT positivity (OR = 0.28, 95% CI: 0.08–0.94, p = 0.04) compared with those with LLIN/HH size ratio <0.5. HH spraying within the previous three months was not significant predicators of malaria prevalence. Similarly sex was not statistically significant malaria-risk factor. In the multivariate model, proximity to mosquito breeding site and LLIN/HH size ratio remained significant predictors of mRDT positivity with OR, 95% CI and p-values of 0.11, 0.12–0.64, 0.014; 0.27, 0.08–0.92, 0.036 respectively. Age and LLIN use the previous night were not significant predictors of mRDT positivity in both univariate and multivariate analyses.

## Discussion

In this study the crude malaria prevalence was 2.8%. The malaria indicator survey 2011 for the entire Amhara Region reported a prevalence of 2.0% [6], Jiga was not particularly sampled during that survey. With enhanced deployment of control interventions malaria reduction is expected, but the asymptomatic carriers in Jiga are sizeable compared to the Regional picture and even nationwide.

Nearly comparable existence of *P*. *falciparum* and *P*. *vivax* was observed in the present study area. This is not in line with the general nationwide paradigm of *Plasmodium* species composition. In Ethiopia, *P*. *falciparum* and *P*. *vivax* make up about 60% and 40%, respectively [12]. However, in some locality like in southwest Ethiopia, the predominance of *P*. *vivax* was reported [13]. In this study, there was no difference in malaria prevalence between age groups and sexes, although this was not statistically tested due to the lower number of mRDT positives. However, the result is similar to a previous study in a different setting [14]. Other studies reported a significantly higher malaria incidence in children than adults in Ethiopia [15] as well as neighboring Somalia [16]. Studies have also shown that the risk of malaria infection varied by sex with some reporting males at higher risk than females [17, 18].

IRS coverage in the previous six months of the study period was 85.5%. This observation could not be compared with previous findings due to scarcity of published records on IRS coverage in the study area proper. But for the entire Amhara region IRS coverage in the last 12 months was estimated to be 53% in 2011 [6] showing the increased application of spraying at least in the study area. However, this study found no statistically significant association between malaria prevalence and house spraying. This is likely to be related with lack of statistical power, since previous studies have demonstrated the protective effect of IRS in Ethiopia and Eritrea [19–21].

In this study, 95.9% of the study HHs had at least one LLIN showing a significant net coverage compared to the status in 2011 which was 73.7% for the Amhara Region [6]. In a nationwide survey in 2005 it was estimated that 6.5% of the Ethiopian HHs had ITN [22]. Troubled by the most recent large scale epidemics in the same year (the most severe and of higher magnitude epidemics was in 2003) in the country [23, 24] the government set an ambitious national goal of achieving 100% ITN coverage in malarious areas of the country with a mean of two ITNs per HH [25] through distribution of about 20 million LLINs by the end of 2007. However, in 2007 the estimated national ITN coverage was scaled-up only to 65.6% [22]. Although the goal was not achieved within the specified period this was a dramatic expansion compared to the baseline in 2005.

Mainly supported by the grant from the Global Fund to fight AIDS, Tuberculosis and Malaria between 2004 and 2007 a total of 17.2 million ITNs were distributed to malarious areas of the country according to FMOH records. A national program distributed a total of over 41 million LLINs between 2004 and 2011 [26]. The most recent Malaria Indicator Survey (MIS), in 2011, showed that LLIN ownership had dramatically increased from the baseline in 2000, but was still below target levels [27]. Up to 2012 than 45 million nets have been distributed in Ethiopia. Free mass distribution, catch-ups, replacement of older nets, etc were being conducted. The above sources show that the goal of the FMoH is to achieve 100% ITN coverage in malarious areas of the country with at least one LLIN per sleeping space to ensure at least 80% of people at risk of malaria use LLINs.

The coverage of LLIN in the current study in 2013 is encouraging and almost near to the FMoH objective though it could still be improved. But LLIN use is limited by socioeconomic factors like house size or bed ownership. It is expected that the number of LLIN per HH is a factor of HH size. But our findings show that this was not always the case. LLIN to HH size ratio was very low in the study area and net was not available for everybody. Out of the 376 HHs having at least one LLIN only 98 had at least one LLIN for two people showing that there were no enough nets for everybody to sleep under in most HHs. If 2 people are assumed to sleep under one net 568 family members did not have access to LLIN out of the total 1851 people in 376 LLIN-owned HHs. The proportion of population with access to LLIN within their HH was 69.3%. Totally there were 692 LLINs (average number of LLINs in each HH is 1.4%). When this was divided by the 1851 people the ratio is 0.4 which means that on average there was less than 1 LLIN for 2 people.

This may explain why malaria prevalence in the current study area didn’t decrease since the Amhara 2011 MIS. So LLIN provision has to be increased taking into account HH size. Secondly, improved socioeconomic development is required so that HHs could accommodate and properly use the provided LLINs. It will be useless if houses cannot accommodate the nets. The current RDT finding suggests that the ongoing control interventions are inadequate to rapidly reduce malaria cases and put Ethiopia among the list of countries whose current agenda is malaria elimination or at least pre-elimination.

Possibly even a higher prevalence rate would have been found if microscopy were used together with the mRDT. Although mRDT is rapid, cheaper and simple to perform in remote rural settings it has several comparative disadvantages [28]. Thus, one major limitation of this study was complete dependence on mRDT. For logistic reasons it was not possible to prepare blood smears. Further, many different molecular assays are available with superior diagnostic performance to both RDTs and microscopy especially to detect submicroscopic infections [29]. Another limitation of this study is that although it is better powered for malaria prevalence estimation it is underpowered for risk-factor analysis.

## Conclusions

Although LLIN and IRS coverage at HH level was encouragingly high in the study area, asymptomatic malaria infection is persistent among the study participants. Carefully-coordinated regular surveillance and response systems must be in place to thoroughly address the impact of ongoing control interventions and associated risk factors in the locality. The findings are believed to contribute towards improving malaria control efforts in Jiga and its surroundings.

## Abbreviations

CI: confidence interval
FMoH: federal ministry of health
HH: household
IRS: indoor residual spraying
ITNs: insecticide treated mosquito nets
LLIN: long-lasting insecticidal net
MIS: malaria indicator survey
mRDT: malaria rapid diagnostic test
OR: odds ratio
